# Salivary Transcriptome and Mitochondrial Analysis of Autism Spectrum Disorder Children Compared to Healthy Controls

**DOI:** 10.3390/neurosci5030022

**Published:** 2024-08-06

**Authors:** Mark Cannon, Ryan Toma, Sri Ganeshan, Emmery de Jesus Alvarez Varela, Momchilo Vuyisich, Guruduth Banavar

**Affiliations:** 1 Ann and Robert Lurie Children’s Hospital, Northwestern University, Chicago, IL 60611, USA; 2 Viome Research Institute, Los Alamos, NM 98011, USAmomo@viome.com (M.V.); guru@viome.com (G.B.); 3 MITOSWAB Religen Labs, Plymouth Meeting, PA 19462, USA; 4 Facultad de Odontología, Universidad CES, Medellin 35 73A-45, Colombia

**Keywords:** autism, transcriptome, mitochondrial, probiotics, xylitol

## Abstract

Autism rates have been reported to be increasing rapidly in industrialized societies. The pathology most often combines neurological symptoms associated with language and social impairments with gastrointestinal symptoms. This study aimed to measure differences in oral metatranscriptome and mitochondrial health between ASD children and neurotypical USA and Colombia (“Blue Zone”) children. In addition, this study aimed to determine whether using prebiotics and probiotics would change the oral microbiome and mitochondrial health of ASD children. Buccal swabs and saliva samples were obtained from 30 autistic individuals (USA) at three intervals: prior to intervention, post-prebiotic, and post-probiotic. In addition, a subject component who were neurotypical, which included individuals from the USA (30) and Colombia (30), had buccal swabbing and salivary sampling performed for metatranscriptomic and mitochondrial comparison. Significant differences were observed in the temporal data, demonstrating shifts that interventions with probiotics and polyols may have precipitated. Particular bacterial strains were significantly more prevalent in the autism group, including a strain that reduced neurotransmitter levels via enzymatic degradation. This supports the hypothesis that the microbiome may influence the occurrence and degree of autism. Verbal skills increased in six of the 30 ASD subjects following xylitol and three more after probiotic supplementation, according to both parental reports and the subjects’ healthcare providers.

## 1. Introduction

Autism spectrum disorder (ASD) is defined as a chronic, inflammatory, and systemic disease, usually with both gastrointestinal and neurological symptoms [1]. In addition to impairments in language and social interactions, ASD children display restricted interests and repetitive behaviors [2]. As is the case with many modern pathological conditions, ASD is considered an epigenetically initiated disease [3,4,5]. The rate of ASD in the population has been estimated to be almost one in 40, representing a marked increase in occurrence [6]. Several factors have been proposed to influence the occurrence of ASD in Western societies [7,8,9,10,11,12]. The foremost concern is that the overuse of antibiotics and/or significant dietary shifts have been detrimental to the long-term relationship between homo sapiens and our symbiotic microbiome [13,14,15,16]. Indeed, all human and microbial genetic structures must be considered within an evolutionary framework [17,18]. We have evolved as a single unit, the holobiont [19]. Modern dietary and hygienic practices may have adversely affected this relationship, resulting in the lack of the “Old Friends” and new pathological relationships with bacterial strains previously not part of the evolved holobiont [20,21,22].

Previous research reported a link between propionic acid-producing bacteria and the development of autistic symptoms [23]. Propionic acid infusions into adult rat cerebral ventricles produce behaviors consistent with autism [24]. These behaviors associated with autism and neuroinflammatory aspects, metabolic effects, and epigenetic changes demonstrated a “central effect” [25,26]. MacFabe et al. reported similar results when delivering propionic acid subcutaneously and then intraperitoneally [23,27]. In addition, propionic acid exposure to human lymphoblastoid cell lines initiated atypical immunological responses [28,29]. However, propionic acid has positive health effects in adults, including cholesterol-lowering effects, anti-inflammatory effects, and weight loss [30]. Calcium propionate is a commonly used food preservative in ultra-high-processed foods, although its use is controversial. For example, a large fast-food restaurant chain announced the discontinuation of calcium propionate due to concerns about behavioral changes in children consuming calcium-propionate-preserved bread [31].

Xylitol is a dietary supplement (classified as an alditol, 5-carbon pentitol) that is natural, not artificial, and can substitute for sucrose. Xylitol occurs naturally in the human diet and is present in fruits, vegetables, and tubers. The liver in a healthy individual generates approximately 15 grams of xylitol daily as a metabolic intermediate linking the critically essential pentose phosphate shunt (5-carbon sugars) and glucuronic acid (detoxification) pathways [32]. Because xylitol is as sweet as sucrose but is low-glycemic, lower in calories, and low-insulinemic, it can substitute for sugar with substantial metabolic benefits in prediabetic and diabetic conditions [33,34].

Xylitol has been extensively studied and demonstrated safe with appropriate use and has notable benefits for preventing dental decay and periodontal disease [35,36]. For over 50 years, xylitol has replaced sugar in foods to reduce tooth enamel decalcification and in the diabetic diet to reduce postprandial blood glucose and insulin extremes [33]. Recent evidence demonstrates that xylitol actively improves oral and systemic health with anti-biofilm, anti-inflammatory, antioxidant, and anti-diabetic properties [32,33]. Excess consumption may have a laxative effect but is well-tolerated after a brief adaptation period. Xylitol’s other reported benefits include the control of blood sugar and obesity, improved skin and wound healing, reduced bone resorption with increased bone strength, and reduced respiratory, sinus, and ear infections [37,38,39,40,41,42]. Xylitol is also reported to inhibit tumor growth in studied cancer cell lines [43,44,45].

In a 2018 study, the gastrointestinal microbiome of 30 autistic 5- to 9-year-old children was quantitated by real-time PCR of stool samples, and the gastrointestinal symptoms were assessed [46]. After the children were supplemented with probiotics, the stool PCR demonstrated increases in Bifidobacteria and Lactobacilli, with significant improvements in the severity of autism and gastrointestinal symptoms compared to their baseline evaluation. A review of 16 articles describing gut microbiome interventions in ASD patients (with antibiotics, prebiotics, probiotics, vitamin supplementation, and fecal microbiota transplantation) demonstrated improvements in behavioral and gastrointestinal symptoms [47]. Another systematic review of 14 articles [48] concluded that prebiotics and synbiotics were efficacious in behavioral symptoms of ASD. In a rat model of autism, supplementation with *Bifidobacterium longum* or fecal matter transplantation restored the calcium propionate acid-induced dysbiosis in a treatment-specific manner [49]. In another animal model, with germ-free mice, microbiota transplantation from typically developing children did not induce tryptophan and serotonin metabolism changes. However, fecal microbiota from ASD children did, and subsequently, the mice developed ASD-like behavior [50]. Finally, a double-blind, randomized crossover clinical trial study showed significant improvements after the administration of probiotics in gastrointestinal symptom scale, maladaptive behaviors, communication skills, and perceived parental stress level [51].

Deep sequencing of the metatranscriptome has provided significantly more data than was previously possible. Approximately 30,000 strains are included in the database. Metatranscriptomics, also known as RNA-seq or RNA sequencing, can examine the quantity and sequence of RNA in a sample using next-generation sequencing (NGS). RNA-seq analyzes the transcriptome of gene expression patterns encoded within RNA [52]. The transcriptome is essential for connecting genomic information with functional protein expression. RNA-seq identifies the genes that are turned on in a cell, their level of expression, and at what time they are activated or turned off [53]. 

The Kyoto Encyclopedia of Genes and Genomes (KEGG) is a collection of databases dealing with genomes, biological pathways, diseases, drugs, and chemical substances [54]. KEGG is utilized for bioinformatics research and education, including data analysis in genomics, metagenomics, metabolomics, and other omics studies; modeling and simulation in systems biology; and translational research in drug development. A review of the KEGG orthology may reveal differences between control subjects from Colombia or the USA and those diagnosed with ASD. Additionally, abnormal metabolites, enzymes, or microbiome shifts may be associated with pathological conditions.

This study aimed to determine the difference in the oral transcriptome and mitochondrial status between children from a “Blue Zone” in Colombia and children from the USA, both healthy and those diagnosed with ASD. The objectives of this study did not include collecting data on cognitive skills, behavioral observations, or gastric symptoms. The long-term intention was to develop a method of objectively diagnosing autism in infancy with oral salivary sampling, allowing for earlier and more effective interventional therapy.

## 2. Materials and Methods

Institutional Review Board approvals were obtained for both the USA and the Colombia component of the study. The IRB requires a power analysis before approval of the protocol. A biostatistician provided the recommended number of subjects (individuals) to be recruited. However, a possible limitation in the number of ASD and “Blue Zone” recruits was considered in the design of this study. In addition, the prebiotic and probiotic intervention was not approved for the neurotypical groups as that would require treatment without the presence of pathology. However, there were no foreseeable adverse effects from either treatment intervention. The ASD group included 30 children aged 6–21 who were sampled at three different intervals: before the intervention, post-xylitol, and post-probiotic. Complete health and dietary histories were obtained after obtaining guardian consent (assent if possible). Three samples were collected from the ASD group: one before the intervention, 60 days after xylitol, and 60 days after the probiotic. At the appropriate visit, the parents received the intervention materials. For the xylitol intervention, the parents were provided with xylitol toothpaste Spry^®^, xylitol suckers/mints, and xylitol mouth rinse (Xlear Inc., American Fork, UT, USA). Due to concerns regarding patient cooperation, the parent was advised to use a xylitol product five times a day. The goal was to “strive for five” exposures as recommended by the manufacturer (five grams daily). For the probiotic intervention, the parents were dispensed ProBiora Pro^®^, a 90-day supply consisting of 2.5 billion CFU of *Streptococcus oralis* KJ3^®^, *Streptococcus uberis* KJ2^®^, and *Streptococcus rattus* JH145^®^, (ProBiora Health^®^ LLC, Tampa, FL, USA). The parents daily gave the probiotic tablet to the ASD child, sometimes by masking it in applesauce. Parents already had strategies for dispensing medications to their children and used what had always worked previously. Parents were to report any difficulties in either intervention to the principal investigator. Any parent unable or unwilling to fully participate was excluded from the study per IRB guidelines.

The sampling consisted of buccal swabs for MITOSWAB (Religen Plymouth Meeting, PA, USA) testing using the manufacturer-supplied materials and saliva samples for metatranscriptomics by VIOME using their supplied materials to determine the entire range of microbial species and their biochemical functions. The Colombian component included 30 children, ages 6–16, considered healthy by the study parameters and within usual behavioral standards, except for one who had received systemic antibiotics for an acute skin infection. Buccal swabbing and saliva sampling were performed only once in this group. The 30 children aged 6–16 in the USA healthy group were matched as well as possible for age and gender. Dietary and health information were also obtained for these two “healthy” groups, as were consents and assents per IRB requirements.

The diet analysis comprised parent interviews and completion of a pediatric diet diary. This was performed for all USA children but not for the Colombian children, who all ate only locally obtained food. The ASD children’s caregivers all reported some dietary intervention, as that is the standard of care for the community. The majority had been placed on a gluten-free diet at an early age by parents/guardians, and all were receiving therapy either in school or local facilities. As is typical with autism, the children were reluctant or resistant to new food introduction. However, the healthy USA children’s parents reported daily fresh fruit and vegetable servings. Over 75% of those parents reported that they avoided ultra-high-processed food and prepared meals fresh. However, parental reporting and the completed diet summary are difficult to substantiate. On the other hand, the majority also reported purchasing organic produce or frequenting local farmer’s markets.

The health history was submitted and approved by the IRB. It was a standard form utilized by many pediatric healthcare professionals. The object of the form was to confirm the diagnosis of autism and to confirm the category of “healthy”. For the purpose of this study, the definition of healthy was a child with no chronic disease. Allergies, or history of prescription medications, were an exclusionary condition. Any medical diagnosis of chronic disease and a history of antibiotics was also exclusionary. Other childhood viral infections, such as the common cold, were not exclusionary. In addition, the child could not have a reported BMI (25–29.9), age-adjusted, over 85%.

The ASD group was predominantly male, consisting of 28 males and two females, with an average age of 11. It was not possible to exactly match the neurotypical groups with the ASD group, the USA neurotypical group comprising 18 males and 12 females, with an average age of 10. In comparison, the “Blue Zone” group consisted of 17 males and 13 females, with an average age of 9.

For the metatranscriptomic analysis, saliva was analyzed using a clinically validated Viome Life Sciences CLIA laboratory test, as previously described (https://www.future-science.com/doi/10.2144/btn-2022-0104). The lab methods are automated and performed on the King Fisher and Hamilton STAR platforms. Samples were lysed using a combination of chemical and mechanical lysis. Total nucleic acids were extracted, and DNA was degraded using the DNase enzyme. Non-informative RNAs (microbial and human ribosomal RNAs and human hemoglobin/myoglobin transcripts) were physically removed using subtractive hybridization. The remaining RNAs were converted to cDNA, labeled with dual unique barcodes, and sequenced on the Illumina NovaSeq 6000 (San Diego, CA, USA) instrument using 2 × 150 paired-end chemistry. 

The taxa were identified using a clinically validated bioinformatic method that uses a catalog of nearly 50,000 microbial genomes with gene annotations, the KEGG Orthology. Reads mapping to the microbial genes were used to quantify KEGG orthologs (KOs). Salivary samples were analyzed at the VIOME Laboratory (Viome Life Sciences Inc., Seattle WA, USA), where the raw data from the RNA sequencing are available. A Spearman correlation and a chi-squared test were conducted on taxa or KO expression data. Benjamini–Hochberg correction was applied to the *p*-value for significance assessment.

MITOSWAB sampling was done to analyze mitochondrial health. Mitochondria are organelles that produce energy to power cells. They produce ATP, the cell’s energy currency required for all bodily functions. They also contain DNA, which makes them highly dynamic. Otherwise known as the “power plants” of the body, their proper functioning is critical to overall health and longevity. The MITOSWAB is a noninvasive test that quantitatively and qualitatively analyzes the mitochondrial electron transport chain (ETC) to evaluate mitochondrial dysfunction. It reports on the functions of complexes I and IV, critical components of the mitochondrial ETC. Citrate synthase activity was measured to quantify the number of mitochondria in each sample. Information regarding mitochondrial function is essential and can assist in developing therapeutic strategies for improving mitochondrial health. The samples were analyzed by Religen Labs (Plymouth Meeting, PA, USA).

## 3. Results

### 3.1. Microbiome Differences

**Figure 1:**
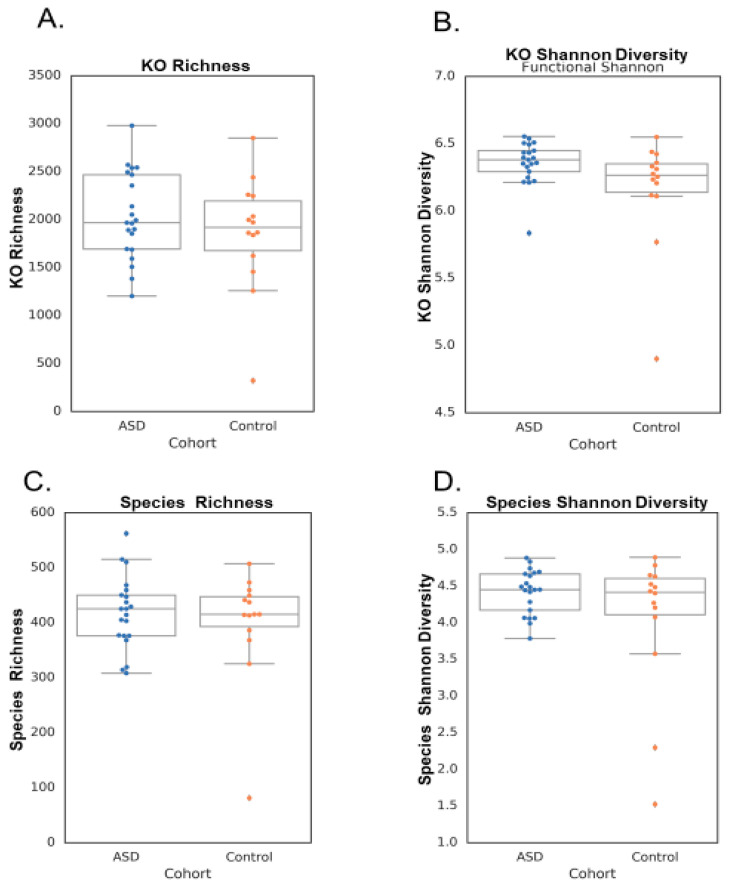
KO and species richness and Shannon diversity between groups. (**A**) KO richness, (**B**) KO Shannon diversity, (**C**) species richness, and (**D**) species Shannon diversity between the ASD and control cohorts. There were no significant differences between the cohorts for any of the sequencing metrics.

Spearman correlations demonstrated significant differences between each participant and the intervention group. Neurotypical controls had 645 unique taxa, the ASD pre-treatment group had 609 unique taxa, the ASD post-xylitol group had 629 unique taxa, and the ASD post-probiotics group had 592 unique taxa. A comparison of the microbial richness and the functional richness of the ASD subjects versus those of the control children is shown in Figure 1 and Table 1.

The “whole foods children” (nickname for healthy children whose parents all reported using organic and fresh foods, with no preservatives or fast food if possible) were all reported to be neurotypical and healthy. The combined groups of ASD versus control were not significantly different in Shannon diversity or function. 

### 3.2. Mictochondrial Differences

**Figure 2:**
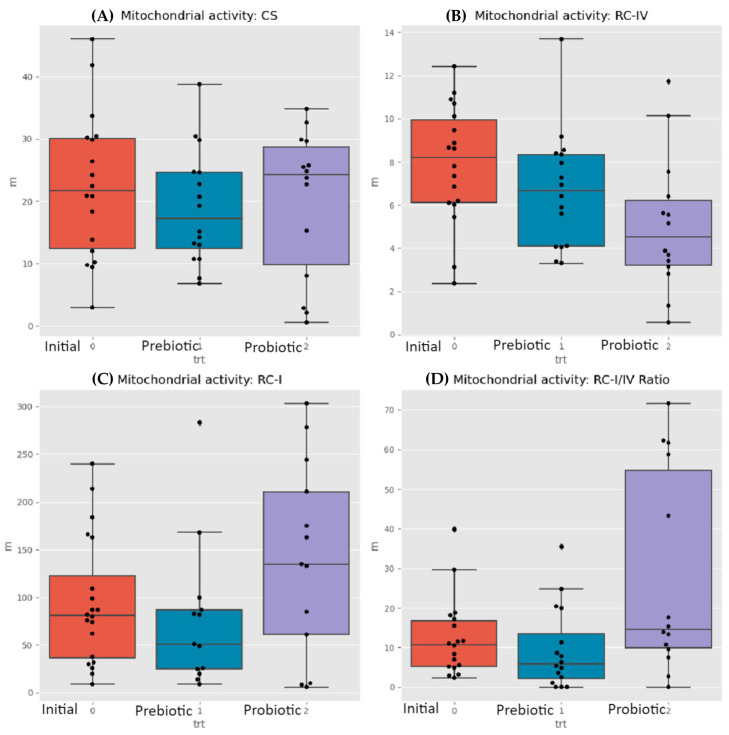
Mitochondrial activity: (**A**) CS; (**B**) RC-IV; (**C**) RC-I; (**D**) RC-I/IV ratio. The orange block represents the initial sampling of the 30 ASD children, the aqua block is after 60 days of prebiotic use, and the purple block is after 60 days of probiotic supplementation. There is no visible change in CS, but RC-IV drops over time. Complex RC-I (and the ratio RC-I/RC-IV) goes down after the xylitol treatment and up after the probiotic is taken. The Wilcoxon test for changes between times detects significant differences (between paired samples of the same users) of the medians of RC-IV and RC-I/IV ratio. Both prebiotics and probiotics affect mitochondrial function. Probiotic intervention enhanced the mitochondrial activity RC I/IV ratio and RC I.

The MITOSWAB samples from Colombia possibly degraded due to the transit time and climate heat. Although the samples were adequately prepared for shipping and delivered to the local FedEx office, their employee accidentally misplaced the package and it was not dispatched promptly. Because the transportation time was extended, the investigators felt that the data obtained may not be accurate. MITOSWAB samples from the neurotypical USA healthy children and the ASD children demonstrated differences in the ETC complexes I and IV. In the control group, four subjects out of 20 (corrected samples, without outliers) had a low ETC complex I, and one out of the 20 had an ETC complex IV defect. The ASD group had seven subjects with a low complex 1, one with a low ETC complex IV, and two with a high complex IV, as seen in Table 2. Significant differences between individuals and groups exist for the ASD groups before intervention, after prebiotics, and after probiotics in the respiratory complex-I-to-IV ratio and respiratory complex IV levels, as shown in Figure 2B,D. The Wilcoxon test for changes between times detected significant differences (between paired samples of the same users) in the medians of the RC-IV and RC-I/IV ratios. Citrate synthase levels (see Figure 2A) did not appreciably change but RC 1 levels appeared to be reduced after prebiotics but increase after probiotics (Figure 2C).

### 3.3. KEGG Orthology 

KEGG orthology (KO) analysis demonstrated that, at baseline, there are 37 significantly different taxa proportions in ASD and healthy groups. Only seven of the 30 patients in the ASD group had higher expression levels. For KOs, these numbers were 27 and 1/27, respectively. Of the seven unique taxa, only one enzyme was expressed significantly more than the others. This enzyme was identified as a hydrolase, N-acyl-D-amino-acid deacylase, produced by a specific bacterial strain typically not found in neurotypical children [55].

**Figure 3:**
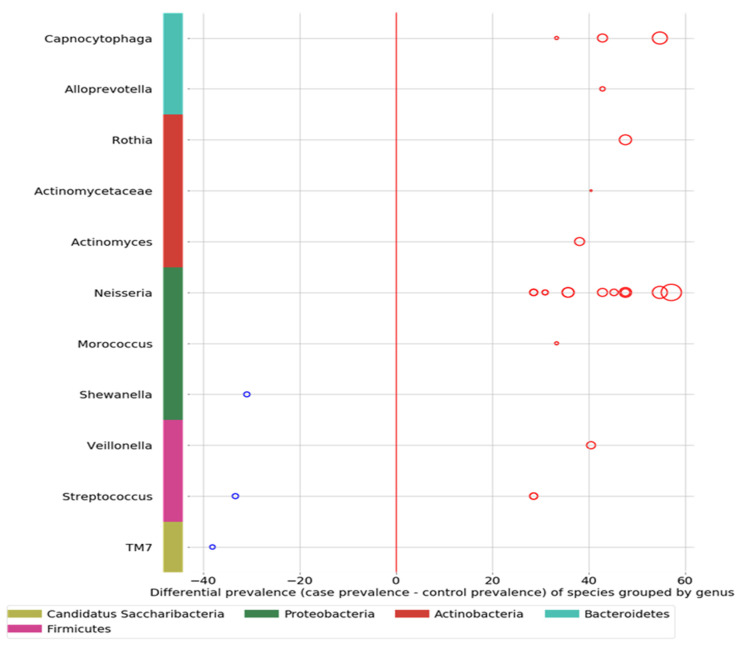
Eleven genera from five phyla show differential prevalence between the ASD and control groups (chi-squared test with *p* < 0.05). A different prevalence of taxa exists between the two groups. The larger the circle, the more pronounced the difference. The red circles represent the up-regulation in 30 ASD subjects, and the blue circles represent the up-regulation in 60 neurotypical children.

**Figure 4:**
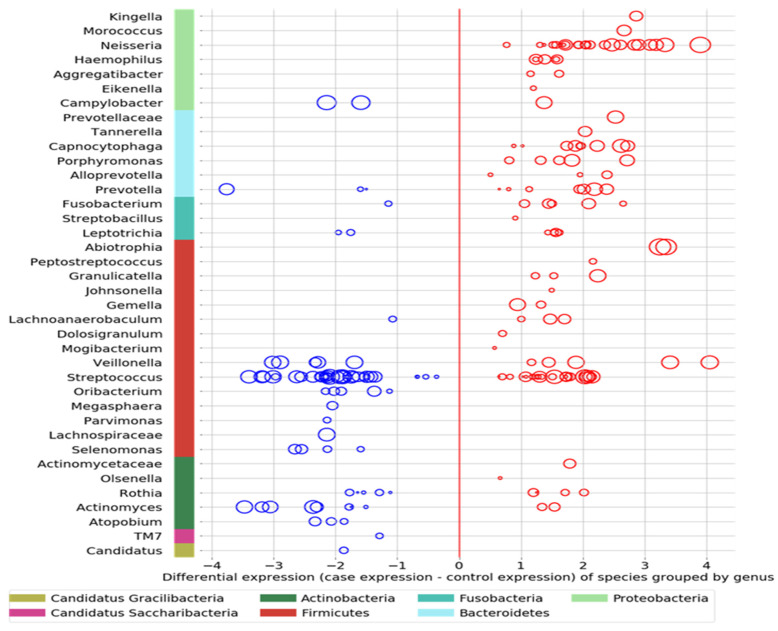
The differential expression of the genus and phylum taxa in the ASD subjects versus the neurotypical “Bule Zone” controls. The oral microbiome of the ASD subjects was significantly different from that of the neurotypical controls. Thirty-eight genera from seven phyla show differential expression between the ASD and control groups (chi-squared test with *p* < 0.05). The ASD group presented as 30 individuals, and the control group was combined with components from the USA and Colombia (60 total). For example, the Streptococcus and Prevotella genera had species and strains that were both up-regulated and down-regulated.

- Comparing the ASD subjects to the neurotypical, different species prevalence between the groups was demonstrated in 11 genera. Specific taxa, such as the Streptococcus genus, had up-regulated and down-regulated strains in the ASD group (see Figure 3). Neisseria, Leptotrichia, Prevotella, Capnoctyophaga, and Fusobacterium genera were up-regulated in the ASD group. The oral microbiome of the ASD subjects was significantly different from that of the neurotypical and “Blue Zone” controls. Species from the genera Treponema, Prevotella, Eubacterium, Selenomonas, Oribacterium, Lachnospiraceae, and Actinomyces were increased in the neurotypical (see Figure 4).

### 3.4. Verbal Skills Development

**Figure 5:**
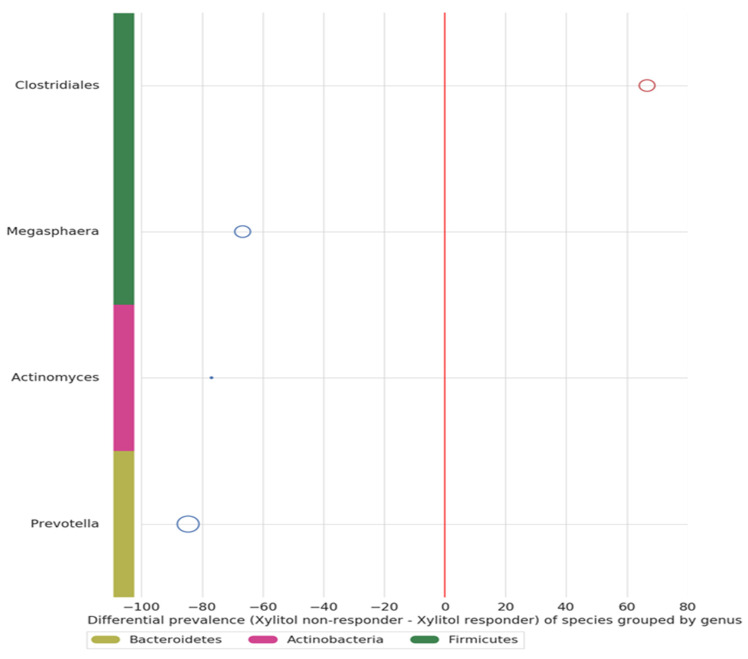
The verbal xylitol responders (six) were a distinct subset of the ASD group (30). Four genera from three phyla showed differential prevalence between the xylitol responders and non-responders (chi-squared test with *p* < 0.05). This may have occurred because of parent–subject compliance, simply due to the prebiotic-specific effects, or even due to the many possible initiating factors of ASD.

Within the timeframe of xylitol exposure, six of the 30 ASD participants demonstrated a significant improvement in verbal skills, becoming verbal after previously not being capable of creating sentences. Because this was an incidental finding, verbal data collection was not quantified before or after the xylitol intervention but only as a comment field in the overall health assessment. This was unfortunate, because a detailed evaluation by a speech pathologist before and after xylitol intervention would have been invaluable. The improvement in verbal skills was significant for all six participants. An additional three participants showed improved verbal skills after the probiotic challenge. Due to the construction of the study protocol, no objective mechanism for speech evaluation was considered. However, both the parents and healthcare staff reported verbal improvements. In addition, three more became more verbal after the probiotic intervention. Bacterial species from three phyla (four genera) were up-regulated in the xylitol responders compared to the non-responders (see Figure 5). The microbiome difference between the later three responders and non-responders is unknown because the study had already completed the IRB-approved sampling and data collection.

## 4. Discussion

A hydrolase, N-acyl-d-amino-acid deacylase, was found to be expressed significantly more in the ASD group. Deacylase catalyzes the endogenous molecules, N-acyl conjugates of amino acids and neurotransmitters (NAANs) [56]. NAANs are significant in information transfer and control of the nervous, vasculature, and immune systems. NAANs are glycine, GABA, or dopamine compounds conjugated with long-chain fatty acids [57]. Interestingly, previous studies have reported that autistic children have decreased GABA levels, and researchers have hypothesized that an imbalance between inhibitory and excitatory signals, such as GABA and glutamate, causes the symptoms of autism [58,59]. Because studies have shown that gut bacteria can significantly alter GABA levels, this enzyme’s effect on NAANs may be responsible for some of the expressions of autism [60]. This would explain why fecal matter transplants have been used to change the gut microbiome in ASD with much-heralded success [60,61]. Interestingly, the oral microbiome has been designated a gateway microbiome, influencing the gut microbiome, placental microbiome, and the blood–brain barrier [62,63]. This would make the oral microbiome’s eubiosis key to the gut microbiome eubiosis. A case in point is that a key oral pathogen has been associated with metabolic syndrome, atherosclerosis, and Alzheimer’s [64,65,66,67]. As used in the study, oral probiotics have documented efficacy against this key oral pathogen, *Porphyromonas gingivalis* [68]. 

Xylitol acts as a prebiotic, shifting the microbiome by inhibiting pathogenic bacteria and viruses [69,70,71]. Recent research into inhibiting virus pathogens by xylitol has stimulated interest due to the COVID-19 pandemic [72]. The effect of dietary xylitol on hRSV infection was investigated in a mouse model, and significant results were reported [73]. The mice received xylitol for 14 days before and three days after viral exposure. The mice receiving xylitol had significantly reduced viral lung titers than the controls receiving phosphate-buffered saline (PBS). Fewer CD3+ and CD3+CD8+ lymphocytes, whose numbers indicate inflammatory status, were reported in the mice receiving xylitol. These results demonstrated improved hRSV infection outcomes and reduced inflammation-associated immune responses to hRSV infection with dietary xylitol. The same researchers previously reported positive effects of xylitol on mice with influenza A virus infection (H1N1), as well as a decrease in recruitment of inflammatory lymphocytes. [74]. The anti-inflammatory and antiviral properties of D-xylose/xylitol in respiratory conditions are the subject of a patent application (number WO1999048361A1) filed in 1998 in the United States [75]. Subsequently, xylitol is the main active ingredient in nasal spray products, such as Xlear Sinus Care.

The xylitol responders were a distinct subset of the ASD group, and several possibilities would explain this. The first possibility is that there are different autisms, some more environmentally sensitive than others, with subjects responding differently due to background settings. For example, Bis Phenol A’s reported role in autism would be environmental [76,77]. However, the role of the microbiome is well documented, and xylitol did shift the microbiome into the “responder” category. The supplements of prebiotics and probiotics both changed the oral microbiome. Strain shifting occurred within the specific genus Prevotella, with some species/strains more or less prevalent in the ASD subjects than in neurotypicals. This may be evolution-driven; as one critical species or strain is reduced, another takes its niche, and the host functions are affected epigenetically by the strain’s metabolites (post-probiotics). Bi-directional functions, functioning in two directions, best explain the relationship between the host and the microgenome. The holobiont consists of the host, the host’s genome, the microbiome, and the microgenome, which is the genome of the microbiome. The holobiont has bi-directional influences; all components attempt to regulate each other to maintain the environmentally determined status quo based on the history of successful integration. Unfortunately, many factors, including newer modern-day interventions that were historically not naturally present, may negatively affect the host’s health.

Autism spectrum disorder (ASD) is reported to be associated with dysbiosis in the oral microbiota. Since the oral cavity is the start of the gastrointestinal tract, this supports the theory of a microbial gut–brain axis in ASD and possibly the existence of a microbial oral–brain axis. Oral bacteria may be transported to the brain through various pathways, including standard dental procedures. The link between the oral microbiome and neural pathologies has been reported in the literature [78]. In contrast, a recent publication in Cell presented the hypothesis that ASD influenced the diet, resulting in a significant change in the microbiome [79]. This article claimed that previous studies were underpowered, too small, and did not adjust for confounders. However, this may be a classic “biofeedback”, whereas dietary preferences may also be driven by the microbiome, such as in ASD, influencing dietary choices [80]. An example would be the decrease in the Rothia genus in subjects diagnosed as autistic in the present study, often with symptoms of gluten intolerance [81,82,83]. Their oral microbiome will not degrade gluten, causing discomfort, and they can then preferentially refuse gluten-rich foods, hence being considered “picky”. The present study included a dietary summary indicating that the healthy USA cohort’s rich and diverse diet influenced their overall health. However, prebiotics and probiotic interventions have provided therapeutic benefits without dietary interventions, which would place diet into a more proper perspective [84,85,86].

A randomized clinical trial recently published reported a significant improvement from baseline in the Vineland-3 Adaptive Behavior composite score (*p*  =  0.03) with probiotic treatment. The authors reported a trend for increased social/geometric viewing ratio following probiotic treatment compared to placebo. Probiotic-associated directional improvements in adaptive behavior measured by Vineland-3 and social preference measured with eye-tracking were noted [87]. Fecal matter transplant treatment for ASD has also been demonstrated to produce significant results in modifying both gut and ASD symptoms, apparently with documented long-term benefits [88]. Microbiome shifts may also have strong epigenetic effects [50,89]. A recently published study of the gut microbiome using REFS identified a set of bacterial taxa that can be used to predict the ASD status of children in three distinct cohorts. Their results support “that the gut microbiome has a strong association with ASD and should not be disregarded as a potential target for therapeutic interventions” [90].

Previously published studies have demonstrated that the oral microbiome of children with ASD is uniquely different from that of neurotypical children. A study with RNA extraction and shotgun sequencing of saliva reported that 12 taxa were changed between the developmental groups, and 28 taxa separated those ASD patients with and without gastrointestinal issues. In addition, five microbial ratios differentiated ASD from control participants (79.5% accuracy), three distinguished ASD from developmental delay (76.5%), and three distinguished ASD children with/without gastrointestinal disturbance (85.7%). The Kyoto Encyclopedia of Genes and Genomes microbial database was utilized to assess taxonomic pathways and compared with a one-way analysis of variance [91]. Studies have also been published reporting a gut microbiome shift in other mental disorders, such as anxiety, depression, bipolar, and schizophrenia. In a study published in Translational Psychiatry, the author’s syntheses identified specific taxa often associated with mental disorders, including lower levels of bacterial genera that produce short-chain fatty acids such as butyrate, higher levels of lactic-acid-producing bacteria, and higher levels of bacteria associated with glutamate and GABA metabolism [92]. Their results were not dissimilar to those of the current study.

This study determined the differences in the oral transcriptome and mitochondrial status between children from a “Blue Zone” in Colombia and children from the USA, both healthy and those diagnosed with ASD. Although the objectives of this study did not include collecting data on cognitive skills, behavioral observations, or gastric symptoms, the verbal skills of six of the ASD children were reportedly improved with prebiotic and an additional three with probiotic intervention. The results should be replicated in more extensive trials to develop a method of objectively diagnosing autism in infancy or young childhood, allowing for earlier and more effective interventional therapy. Correcting the oral microbiome dysbiosis with prebiotics and probiotics may positively influence the gut microbiome, as reported in previous studies, and reduce the neurotransmitter imbalance that may arise in a more industrialized society consuming ultra-high-processed foods.

## 5. Conclusions

The transcriptomes of children with ASD in the USA are different from those of healthy children in a developing country and neurotypical children in the USA. Intervention with prebiotics (e.g., xylitol) and probiotics may significantly affect the oral transcriptome.

## Figures and Tables

**Table 1:** Microbial and Functional Differences Statistical Analysis.

Metric	Test *p* Value	ASD Mean +/− Std	Control Mean +/− Std
**Microbial Richness**	0.491677	418.43 +/− 65.60	398.79 +/− 101.95
**Functional Richness**	0.32413	2035.14 +/− 453.53	1857.29 +/− 597.31
**Microbial Shannon**	0.113801	4.42 +/− 0.30	4.05 +/− 0.98
**Functional Shannon**	0.056327	6.36 +/− 0.16	6.16 +/− 0.41

**Table 2:** Control neurotypical children’s MITOSWAB results versus ASD children’s, demonstrating differences in ETC complexes I and IV.

Subjects	ETC Complex I Defect	ETC Complex IV Defect
ASD children	7	3
Neurotypical controls	4	1

## Data Availability

Data were protected due to the need to maintain strict privacy of the subjects (children), and the IRB-approved consent contained specific language ensuring complete safeguarding measures. All data are on file at MITOSWAB and VIOME.
